# Unveiling the Evidence for the Use of Pulses in Managing Type 2 Diabetes Mellitus: A Scoping Review

**DOI:** 10.3390/nu15194222

**Published:** 2023-09-30

**Authors:** Daniel J. Thomas, Mojtaba Shafiee, Matthew G. Nosworthy, Ginny Lane, D. Dan Ramdath, Hassan Vatanparast

**Affiliations:** 1 Caribbean Institute for Health Research, The University of the West Indies, Mona, Kingston 7, Jamaica; daniel.thomas03@uwimona.edu.jm; 2 School of Public Health, University of Saskatchewan, Saskatoon, SK S7N 4Z2, Canada; 3 College of Pharmacy and Nutrition, University of Saskatchewan, Saskatoon, SK S7N 4Z2, Canada; mojtaba.shafiee@usask.ca (M.S.); matthew.nosworthy@agr.gc.ca (M.G.N.); 4 Guelph Research and Development Centre, Agriculture and Agri-Food Canada, Guelph, ON N1G 5C9, Canada; 5 Margaret Ritchie School of Family and Consumer Sciences, University of Idaho, Moscow, ID 83844, USA; vlane@uidaho.edu

**Keywords:** pulses, pulse consumption, type 2 diabetes mellitus, glucose, insulin

## Abstract

Management of type 2 diabetes mellitus (T2DM) is a pressing global healthcare challenge. Innovative strategies that integrate superior medical and nutritional practices are essential for holistic care. As such, pulse consumption is encouraged for its potential benefit in reducing hypercholesterolaemia, dyslipidaemia, and triglyceride levels, as well as enhancing glycaemic control. This scoping review aims to assess the depth of evidence supporting the recommendation for pulse consumption in T2DM management and to identify gaps in the existing literature. We conducted a comprehensive search across the databases MEDLINE, Global Health, EMBASE, CINAHL, Web of Science, and the Cochrane Library (up to July 2023). We included population-based studies of any design, and excluded review-style articles. Articles published in languages other than English were also excluded. From the 2449 studies initially identified, 28 met our inclusion criteria. Acute postprandial trials demonstrated improved glucose responses and enhanced insulin responses to pulse-based intervention. Meanwhile, long-term trials reported meaningful improvements in T2DM indicators such as haemoglobin A1C (HbA1c), fasting glucose, fasting insulin, C-peptide, and markers of insulin resistance like homeostatic model assessment (HOMA). Integrating more pulses into the diets of diabetic individuals might offer an efficient and cost-effective strategy in the global initiative to combat T2DM.

## 1. Introduction

Diabetes imposes a substantial global health and economic burden. According to the 2021 Diabetes Atlas, approximately 10.5% of the world’s adult population (equivalent to an estimated 537 million individuals) are currently affected by type 2 diabetes mellitus (T2DM). This number is projected to escalate to 783 million by the year 2045, an increase of about 46% [1]. This growth in diabetes prevalence will likely lead to a pronounced economic impact, with associated costs in 2021 amounting to USD 966 billion and a projected surge to about USD 1.05 trillion by 2045 [1]. The rise in diabetes incidence and prevalence in the 21st century can be attributed primarily to lifestyle changes, including reduced levels of physical activity and dietary shifts such as increased consumption of energy-dense, highly processed, nutrient-poor foods, as well as sugar-sweetened beverages [2]. There is also disparity in diabetes prevalence, with higher rates among low-resource, marginalized communities. Remarkably, three out of four individuals with diabetes reside in low- and middle-income countries [1], where primary and secondary care facilities often suffer from inadequacies due to socioeconomic disadvantage [3]. In the Caribbean, the estimated overall prevalence of diabetes mellitus stands at approximately 9% [4], accounting for 13.8% of all adult deaths in the region [5]. Notably, the North America/Caribbean Region shoulders the highest total diabetes-related health expenditure at USD 415 billion, representing 42.9% of total global diabetes-related health expenditure in 2021 [1].

Effective diabetes management aims to achieve normal glycaemic control through the implementation of dietary and lifestyle modifications alongside the judicious use of anti-hyperglycaemic medication. According to the American Diabetes Association [6] and Canadian clinical practice guidelines [7], diabetes management through dietary strategies involves reducing refined carbohydrates, increasing intake of whole, unprocessed foods, incorporating more fibre, and increasing consumption of pulses. Pulses, defined as the dry, edible seed of the leguminous plants, have been integral to various cuisines for centuries due to their versatility and nutritional benefits. They serve as a rich dietary source of protein, along with soluble and insoluble fibre [8,9]. As dietary guidelines increasingly endorse plant-based eating patterns, it becomes imperative to ascertain the direct impact of pulses in the management of chronic diseases such as diabetes. It is also noteworthy that pulse crops serve as carbon sinks and play a key role in the mitigation of greenhouse gas emissions [10]. They also have symbiotic relationships with nitrogen-fixing bacteria, responsible for the fixation of inorganic nitrogen compounds essential to life [11].

The available evidence suggests that pulses play a beneficial role in mitigating T2DM and other chronic diseases [9,12]. While the nutritional benefits of pulses and their potential role in mitigating T2DM have been explored, a comprehensive synthesis of the recent literature consolidating this scattered evidence is lacking. Further, the existing literature fails to offer a geographical perspective. Understanding where the most significant research efforts are situated compared to diabetes prevalence can provide a unique insight into research priorities and potential biases. Additionally, the mechanisms by which pulses impact T2DM remain underexplored. Given the breadth and diversity of the evidence, we opted for a scoping review over a systematic review. A scoping review allows for a broader examination of the field, encompassing diverse study designs and methodologies, making it ideal for offering a holistic view of the topic, pinpointing gaps in the research landscape, and charting out areas for future systematic exploration. This scoping review primarily aims to comprehensively evaluate the existing body of literature concerning the impact of pulses on the management of T2DM in type 2 diabetic patients. By synthesizing available recent data, this review seeks to provide a holistic understanding of the benefits that pulses offer in the context of managing T2DM.

## 2. Materials and Methods

This scoping review was conducted systematically using a predefined protocol based on the methodological framework established by Arksey and O’Malley [13]. This approach involved identifying the research question, selecting pertinent studies addressing the research question, and organizing and reporting the findings. We adopted the Population (P), Concept (C), and Outcome (O) approach to structure our search strategy and determine eligibility criteria (as shown in Table 1). The research question driving this approach was “What is the extent of evidence supporting the recommendation of pulse consumption (Concept) in the management (Outcome) of T2DM in type 2 diabetic patients (Population)?”.

The review was conducted in accordance with the Preferred Reporting Items for Systematic Reviews and Meta-analyses extension for Scoping Reviews (PRISMA-ScR) guidelines [14]. Eligible sources for inclusion in the scoping review needed to provide specific information regarding pulse consumption in relation to the management or control of Type 2 diabetes in human subjects. The inclusion criteria, outlined in Table 1, were applied. Peer-reviewed journal articles published in English were considered for inclusion.

### 2.1. Study Selection

A systematic search of available peer-reviewed literature was conducted and further refined through collaborative discussions among the authors. The following databases were comprehensively searched from their inception to July 2023: MEDLINE, Global Health, and EMBASE via Ovid, CINAHL via EBSCO, Web of Science via Clarivate, and the Cochrane Library. The finalized search strategy used for Ovid MEDLINE is provided in Supplementary Table S1. Upon solidifying the list of keywords and MeSH (Medical Subject Heading) descriptors, similar searches were performed on the remaining scientific literature databases. All eligible studies that met the inclusion criteria were considered.

### 2.2. Screening Process

The initial search identified 4396 records. These results were imported into Rayyan (available at: https://rayyan.ai/ (accessed on 18 July 2023)) [15] and duplicate sources were removed, leaving 2449 records. Independent screening was conducted by two reviewers (D.J.T. and M.S.). An initial assessment of titles and abstracts, guided by an eligibility form, resulted in the exclusion of 2408 records. Included records (*n* = 41) underwent subsequent full-text screening. Reviewers met during the screening process to share their insights and resolve any conflicts. All disagreements between reviewers were resolved through consultation with a third reviewer (M.G.N.).

**Figure 1:**
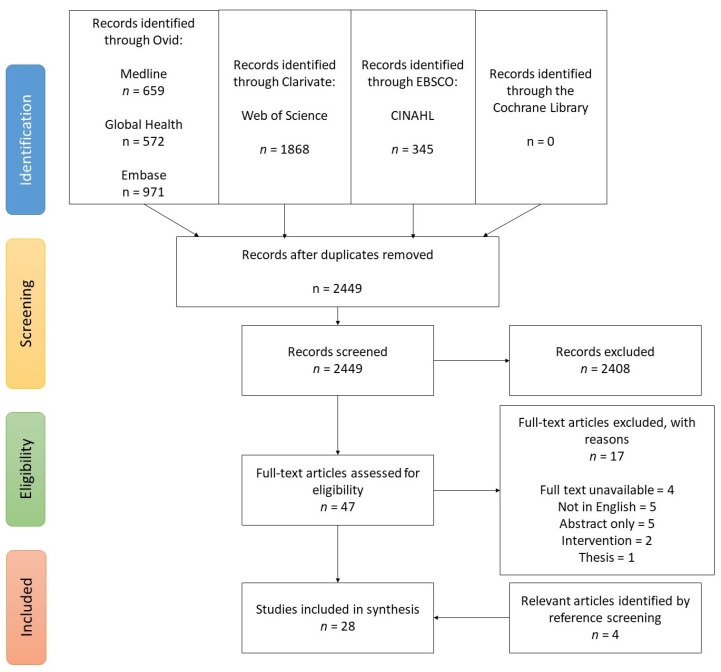
Preferred Reporting Items for Systematic Reviews and Meta-Analyses (PRISMA) reporting flow diagram showing 4-stage article selection process used to identify articles on the effect of pulses in the management of T2DM.

All included studies were additionally hand-searched for references that might have been missed in the initial search. In total, 28 articles proceeded to the data extraction stage. The PRISMA Flow diagram (Figure 1) provides a visual representation of the journey undertaken to compile the final list of eligible sources, alongside reasons for exclusion.

### 2.3. Data Extraction and Synthesis

Table 2 and Table 3 provide an overview of the characteristics of each included study.

## 3. Results

### 3.1. Characteristics of Included Studies

**Figure 2:**
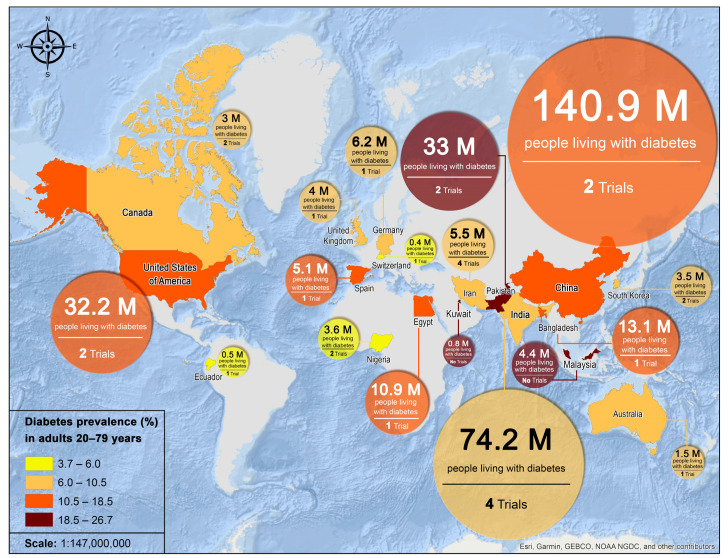
Geographical distribution of trials investigating pulse consumption in T2DM management, alongside corresponding diabetes prevalence and the number of individuals living with diabetes. Data on diabetes are derived from the International Diabetes Federation (IDF) Diabetes Atlas, 10th edition, 2021 [44].

The comprehensive search strategy identified 4396 articles, and after removing duplicates, 2449 articles remained. Ultimately, a total of 28 peer-reviewed articles met the inclusion criteria to be included in the scoping review (Figure 1). While it may be assumed that ethical approval was obtained for all human studies included in the review, of the 14 RCTs, 10 indicated approval by an Ethics/Institutional Committee/Review Board, while 8 acute postprandial trials reported similar approval. Among the 28 articles meeting the eligibility criteria, their geographic distribution was as follows: 7 studies were conducted in South Asia (encompassing India, Pakistan, Bangladesh); 4 studies were situated in East Asia (specifically China, Republic of Korea); 9 studies were undertaken within Europe or among European-descent populations (spanning Australia, Canada, UK, Germany, Spain, Switzerland, USA); 2 studies were conducted in Nigeria, and 1 study each originated from Egypt and Ecuador. Iran had the highest number of publications, with several linked to the “Therapeutic Lifestyle Changes” project; however, only 4 of these were translated into English and thus were eligible for this review. Figure 2 illustrates the geographical distribution of the trials included in this review and the corresponding prevalence of diabetes.

In the studies reviewed, a diverse array of legumes was tested both in their natural form and as part of composite meals or foods such as breads or biscuits. Each type of legume has its unique nutritional profile, which might influence its glycaemic impact. Out of the 28 studies in this review, the most frequently studied pulses included lentils (*Lens culinaris*/*Lens esculenta*), chickpeas (*Cicer arietinum*), varieties of beans like pinto, black, white, navy and red kidney (*P. vulgaris*), black-eyed peas, cowpeas (*Vigna unguiculata*), mung beans (*Vigna radiata*), black gram or mash (*Vigna mungo*), adzuki beans (*Vigna angularis*), lab-lab beans (*Lablab purpureus* L.), pigeon peas (*Cajanus cajan*), and green peas (*Pisum sativum*). A significant number of long-term studies were nonspecific about the varieties they studied, often using general terms like “legumes” or “pulses.” Studies from Ecuador [26] and Egypt [32] explored the effects of lupins, while lupin-enriched wheat products were the focus of an Australian study [41].

### 3.2. Outcome Measures

Among the 28 studies included, 14 were acute postprandial trials [16,17,18,19,20,21,22,23,24,25,26,27,28,29] that investigated the short-term or immediate effects of pulse consumption on glycaemic indices (Table 2). The most commonly assessed outcomes in these acute postprandial trials included fasting blood/plasma glucose levels, incremental blood glucose responses, and area under the response curve (AUC) for blood glucose, followed by fasting insulin and incremental insulin responses. Additionally, two of these studies assessed the gastric inhibitory peptide/glucose-dependent insulinotropic polypeptide (GIP) [17] and C-peptide [29].

The remaining 14 studies were long-term trials [30,31,32,33,34,35,36,37,38,39,40,41,42,43] that focused on the impact of pulses and pulse-based diets on glycaemic and insulinaemic parameters in individuals with type 2 diabetes (Table 3). These long-term investigations evaluated changes in fasting blood glucose (FBG), haemoglobin A1c (HbA1c) levels, and insulin resistance using the Homeostatic Model Assessment for Insulin Resistance (HOMA-IR) method. Further, some studies also assessed C-Peptide levels [36], 24 h urinary glucose [30], glycated albumin [39], and fructosamine [33]. The studies evaluated various diets, with some focusing exclusively on pulse foods, while others explored mixed diets that combined pulses with cereals, rice, bread, or potatoes. Additionally, a number of studies pursued a comparative approach, evaluating the effects of diets where pulses were substituted for meat sources [40,42]. Several studies also looked at the effects of innovative pulse preparations on blood glucose parameters. For instance, researchers investigated the effects of bean flakes [17], khichadi (a steam-cooked savoury preparation) made from a cereal-pulse mix [21], roasted flour mix [37], and lupin-enriched cereals such as Weetbix^TM^, multigrain pasta, and bread [41]. Another study delved into the potential benefits of an extruded adzuki bean-based convenience food [39].

### 3.3. Acute Postprandial Trials

Fourteen studies [16,17,18,19,20,21,22,23,24,25,26,27,28,29] evaluated the glycaemic indices of standardized carbohydrate portions from single pulse meals and pulse-based mixed meals (Table 2). These were compared to meals comprised of refined rice, bread, or an oral glucose tolerance test (OGTT) drink, or to mixed meals of bread and eggs. In these trials, initial blood samples were collected for a baseline FBG measurement. Subsequent samples were taken at intervals of 30 min or 1 h, and then periodically, with the shortest duration being 90 min [26] and the longest extending up to 6 h [27] post-consumption of the pulse food or control.

Pulses and pulse-based mixed meals consistently demonstrated lower glucose responses compared to carbohydrate-matched controls, resulting in significantly reduced serum glucose levels over various postprandial time intervals (Table 2). Jenkins et al. [16] measured the glycaemic responses to 15 different foods and reported that type 2 diabetics had significantly lower blood glucose responses to kidney beans, chickpeas, and lentils compared to all other non-leguminous foods tested. Similarly, black-eyed peas and pinto beans had overall lower glycaemic responses than other meals, with the exception of spaghetti for black-eyed peas and spaghetti and rice for pinto beans [16]. Akhtar et al. [18] examined the glycaemic responses of traditional Pakistani leguminous seeds in mixed meals among both nondiabetic and diabetic males. These meals, reflecting customary dietary practices in Pakistan, were compared to those where bread was the main carbohydrate source. It was found that meals with legumes such as grams, lentils, moong (*Phaseolus mungo*), and mash (*Phaseolus aureus*) led to lower blood glucose responses than those dominated by bread [18]. Mani et al. [20] explored the glycaemic and triacylglycerol responses of diabetes patients to five conventional Indian meals based on semolina and different pulses. They found that only specific meals, such as steam-cooked semolina and semolina combined with certain pulses, demonstrated significantly lower postprandial blood glucose (PBGR) responses compared to a glucose load [20]. In 1994, Mani and team [21] further studied glycaemic and triglyceride responses to the traditional cereal-pulse blend called ‘khichadi’. This test meal showed not only a low mean GI value but also significantly reduced postprandial glucose and triglyceride responses [21]. Thompson et al. [24] examined the glycaemic response to traditional meals made of beans and rice in adults with type 2 diabetes. In this randomized crossover trial involving 17 participants, meals were either white rice alone or combined with pinto, black, or dark red kidney beans. The results demonstrated that adding beans to rice considerably decreased postprandial glucose levels at 90, 120, and 150 min compared to rice by itself. Specifically, meals containing pinto and black beans showed a marked drop in the incremental AUC values relative to the rice-only control [24].

Alegbejo and Ameh [25] studied the blood glucose response and glycaemic index (GI) of a rice/bean meal in both type 2 diabetic and nondiabetic participants. For diabetic participants, blood glucose responses to meals of rice/beans were significantly reduced between 30 to 150 min post-consumption compared to 50 g glucose. However, a rice/bean meal did not exert a similar significant impact on the blood sugar levels in nondiabetic participants. Olopade et al. [28] explored the glycaemic responses triggered by different local bean varieties (*Vigna unguiculata* (Linn Walp)) in those with T2DM. Over 12 weeks, they evaluated responses to three bean types: “*oloyin*”, “*drum*”, and “*sokoto white*”. For T2DM individuals, the “*Oloyin*” bean meal gave the highest 2 h postprandial glucose and peak plasma glucose levels, yet had the lowest figures for the maximum increase in plasma glucose from baseline (MIPG). In contrast, the “*drum*” bean meal recorded the peak values for MIPG and incremental area under the glucose curve (iAUGC), signifying its considerable glycaemic effect [28]. Khan and colleagues [23] examined the glycaemic responses of several traditional leguminous dishes commonly consumed in Pakistan, including Mash, Moong, Masoor, Chana dhal, and Biryani, all served with white boiled rice. Their study involved both nondiabetic and diabetic participants, who were administered 50 g carbohydrate portions. When compared against the glycaemic response of chapatti combined with egg (set as 100%), the dishes displayed varying glycaemic responses in both the diabetic and nondiabetic groups. This led the researchers to conclude that when integrated with white boiled rice as part of a composite meal, these traditional leguminous dishes may have limited efficacy in the dietary management of diabetes [23]. While acknowledging the conclusions of these researchers, it should be noted that the inclusion of egg protein in the control meal may confound these results as protein-rich foods, such as eggs, can play an important role in regulating blood sugar levels and improving glucose control [45]. However, when considering the benefit of pulses, the specific combinations and preparation methods should be noted, as not all leguminous dishes offer the same benefits to glycaemic regulation.

Beyond the immediate or acute effects on blood glucose, pulse consumption also has a noteworthy influence on insulin levels [17,19,22,26,27,29]. Tappy et al. [17] investigated the effects of a 50 g starch meal prepared with pre-cooked instant bean flakes on glucose, insulin plasma levels, and glucose oxidation rate in healthy and type 2 diabetic subjects. The study showed that compared to an equivalent potato-based meal, the bean flakes resulted in a significantly lower rise in plasma glucose and insulin levels in healthy subjects within the first 30 min after ingestion. When tested on diabetic patients, the bean flakes meal also demonstrated only moderate elevations in plasma glucose, insulin levels, and glucose oxidation rate [17]. Viswanathan et al. [19] investigated the glycaemic and insulin responses to various legume preparations, termed ‘adai’, in both type 2 diabetic patients and nondiabetic controls. The legumes in the study included Bengal gram, black gram, green gram, red gram, and lentils. The findings revealed that all legumes elicited low glycaemic responses in nondiabetic controls and even lower responses in diabetic patients. In nondiabetic controls, the legumes generally led to lower plasma insulin responses compared to glucose. Conversely, in the diabetic subjects, insulin responses to the legumes were similar to those induced by glucose [19]. In their research, Schafer et al. [22] delved into the effects of meals containing dried peas and potatoes on postprandial glucose and insulin levels of type 2 diabetic patients. Their data suggested that dried peas, being a low-glycaemic and high-fibre food, led to smaller increases in plasma glucose and insulin concentrations compared to potatoes. Moreover, when compared to meals in which potatoes were the sole carbohydrate source, meals of an equivalent carbohydrate serving of peas and potatoes in a carbohydrate ratio of 1:2 yielded lower glycaemic responses [22].

In a phase II clinical trial [26], the impact of cooked *Lupinus mutabilis* and its alkaloids on blood glucose and insulin levels were examined in volunteers with T2DM. Results indicated that both treatments lowered glucose levels, with the cooked form showing a more pronounced decrease. Olmedilla-Alonso et al. [27] studied the effects of two Spanish bean varieties, ‘Almonga’ and ‘Curruquilla’, on glucose and insulin levels in type 2 diabetics. Compared to white wheat bread, both beans led to significantly lower glycaemic responses in the initial 2 h. Glucose peaks were three times lower with beans, and while ‘Almonga’ induced peak insulin levels at 60 min’ ‘Curruquilla’ did so at 90 min [27]. Another study investigated the effects of a bean-based breakfast versus a white rice-based breakfast on postprandial glucose and insulin levels in Chinese patients with T2DM [29]. The results showed that the bean-based breakfast led to significantly lower postprandial glucose levels at certain time points compared to the white rice breakfast, and also promoted higher insulin secretion. Moreover, participants who consumed the bean-based breakfast displayed an improvement in HOMA indices, indicating enhanced insulin sensitivity and possibly improved beta-cell function. Additionally, C-peptide levels were more pronounced in the bean breakfast group, suggesting an increase in endogenous insulin secretion [29]. Considering the above research findings, it is evident that various legumes, including beans, peas, and other pulses, have a significant effect on modulating postprandial glucose and insulin responses in individuals with T2DM.

### 3.4. Long-Term Trials

Fourteen long-term randomized controlled clinical trials [30,31,32,33,34,35,36,37,38,39,40,41,42,43] have been conducted to assess the impact of pulses and pulse-based diets on glycaemic regulation among individuals with T2DM (Table 3). These studies varied in their design, ranging from parallel (P) to crossover (C) trials, and have utilized different interventions, from high-legume and -cereal diets to specific pulse preparations like roasted chickpeas or legume-enriched foods. The durations of these trials spanned from as short as 1 week to as long as 16 weeks, examining a range of outcomes, including FBG, insulin levels, HbA1c, and HOMA-IR (Table 2 and Table 3).

A majority of the trials suggest that incorporating pulses and legumes into the diet can positively influence glycaemic markers, such as glucose levels [30,31,32,33,34,35,36,37,38,39,42] and glycated products like HbA1c [31,32,34,36]. In a crossover clinical trial by Shams et al., 30 type 2 diabetic patients were separated into two groups. One group incorporated 50 g of cooked lentil and 6 g of canola oil into their breakfast, replacing 30 g of bread and 20 g of cheese, while the other group continued with their normal diet. After a 6 week period and a subsequent 3 week washout period, the diets of the two groups were swapped for another 6 weeks. The results indicated that while serum fructosamine remained unaffected in the treatment group, there was a significant reduction in FBG levels [33]. A Bangladesh-based study created a composite flour mix using whole wheat, maize, Bengal gram (desi variety), and lablab bean, at a ratio of 35:15:25:25 of total weight flour mix. The aim was to assess the impact of bread from this mix on T2DM patients. A total of 30 T2DM patients were recruited, with the experimental design spanning 56 days. The main differentiation between the intervention and control diets was the fibre content, being 40 gm/d for the intervention group and 25 gm/d for the control group. Postprandial blood glucose levels after consuming the composite flour bread were significantly lower at all time points when compared to the conventional wheat bread; further after 28 days of intervention FBG was significantly lower in the treatment group [37]. In another study, Sekar et al. introduced twenty stable T2DM patients to a modified diet comprising 75% pulses and 25% cereals for three months. This diet was consumed in the form of idli or dosa at least four times a week. When compared to a control group on a 75% cereal and 25% pulse diet, the modified diet group exhibited a significant drop in HbA1c levels, indicating potential glycaemic control benefits [31]. A randomized trial with 121 type 2 diabetes participants compared a low-GI diet emphasizing legume consumption with a high-wheat-fibre diet over three months. The main outcome was an HbA1c value change. The legume-rich diet resulted in substantial HbA1c level improvements and reduced postprandial blood glucose levels, suggesting enhanced short-term glucose management compared to the wheat-based diet [34].

A cross-over study at Tehran’s Taleghani hospital explored two therapeutic lifestyle change (TLC) diets’ impacts on cardiometabolic factors in T2DM patients. Participants were assigned either a legume-free TLC diet or a legume-based diet, substituting red meat with legumes thrice weekly. Both diets led to significant reductions in FBG, insulin, triglycerides, LDL cholesterol, and total cholesterol. However, the legume-based TLC diet had a more pronounced effect [38]. At the National Research Center in Cairo, researchers examined the effects of traditional plants, like wheat, fenugreek, lupine, and chickpea, on blood glucose regulation in type 2 diabetic patients. Ninety-four participants were divided into nine groups, with eight of them replacing part of their breakfast bread with the test foods for a week. The control group maintained a low-calorie balanced diet. The study observed notable declines in mean blood glucose levels at various time points during a glucose tolerance test, especially with certain foods. Moreover, the study reported significant decreases in serum insulin levels after 2 h for lupine and partially decorticated wheat, while HbA1c data showcased a reduction for most groups, with significant reductions observed for whole wheat and biscuit 1 [32]. Simpson et al. embarked on a 12-week clinical trial with 18 type 2 diabetics and 9 insulin-dependent diabetics (IDDM). After an initial 2-week briefing, participants were placed on one of two diets for 6 weeks, then underwent a 24 h metabolic profile in a hospital. After, they switched diets for another 6 weeks, ending with another metabolic assessment. Type 2 diabetic patients on a diet high in leguminous and cereal fibre displayed significantly lower plasma glucose levels throughout the day compared to the traditional diabetic diet. HbA1c levels, however, remained consistent on both diets [30]. In Shiraz, Iran, a single-centre randomized clinical trial at the Shahid Motahhari endocrinology clinic involved 75 patients with T2DM. Over 8 weeks, participants were divided into one of three dietary groups: one consuming red meat, another consuming soybeans, and the last consuming non-soy legumes. Primary outcomes revolved around fasting insulin, HbA1c, and various cardiometabolic factors. The results, however, showed negligible differences in most cardiometabolic factors from the start to the end among the study groups [40]. These studies underscore the potential advantages of incorporating legumes and pulses into the diets of type 2 diabetes patients. While individual study results may vary, there is a consensus suggesting that these foods can aid in glycaemic control.

While most studies indicated a simultaneous change in insulin levels accompanying alterations in blood glucose [32,35,36,38,39,42], Simpson et al. (1981) observed variations in blood glucose without significant associated changes in insulin levels [30]. Several studies have also investigated the impact of pulse consumption on HOMA-IR scores, an indicator of insulin resistance [35,36,39,41,42,43]. In a controlled trial from Yonsei University, researchers assessed the impact of whole grains and legumes versus refined rice in relation to the APOA5 -1131C variant status of patients with impaired fasting glucose or newly diagnosed type 2 diabetes. Of the 185 Korean participants, those undergoing a 12-week dietary intervention with whole grains and legumes displayed significant reductions in serum glucose, insulin, and HOMA-IR levels, irrespective of their genetic variant. However, those with the C allele of the APOA5 -1131 T > C polymorphism showcased a more pronounced decrease in insulin when on the whole grain and legume diet [35]. Another research project delved into the metabolic effects of replacing refined rice with whole grains and legumes in individuals with impaired fasting glucose or new-onset type 2 diabetes. Over 12 weeks, the group that integrated whole grains and legumes into their diet showed substantial improvements in fasting glucose, insulin, HOMA-IR index, and HbA1c levels. In contrast, the group consuming primarily refined rice witnessed increased glucose and HbA1c levels [36]. In a 4-week randomized trial, 120 type 2 diabetic patients from the Pinggu Hospital of Traditional Chinese Medicine received either a traditional diabetic low-GI diet or a diet featuring extruded adzuki bean convenience food (EABCF). Although both groups noted a decrease in HbA1c and other glycaemic markers, subgroup analysis based on the HOMA indices revealed subtle differences in insulin resistance and sensitivity patterns, though they were not statistically significant [39]. A randomized trial contrasted the effects of two hypocaloric diets—the DASH diet and a legume-based DASH diet—on glycaemic parameters in type 2 diabetes patients. Remarkably, those on the legume-based DASH diet displayed a more substantial decrease in fasting plasma glucose (FBG) than those on the standard DASH diet. A reduction in insulin response was observed at week 16 in both hypocaloric dietary interventions. Additionally, insulin resistance, as measured by HOMA-IR, improved more significantly in the legume-based DASH diet group [42].

A study at the Pennington Biomedical Research Center over 8 weeks involved 36 participants with insulin resistance, aged 18–60, and compared diets rich in white potatoes or pulses. Results revealed that the glycaemic responses of both diets were equivalent within predefined margins. Despite an initially higher glucose AUC in the bean diet group at baseline, both dietary interventions failed to produce significant reductions in fasting glucose concentrations compared to baseline. However, the bean diet led to a notable reduction in insulin response, making it comparable to the potato diet. Furthermore, HOMA-IR displayed significant reductions in both diets [43]. Lastly, an 8-week trial set out to evaluate the effects of lupin-enriched foods in people with moderate-to-well-controlled T2DM. Participants either consumed lupin-enriched or energy-matched wheat-based foods, targeting a replacement of roughly 20% of their daily caloric intake. Contrary to other findings, the short-term consumption of lupin-enriched foods did not yield significant improvements in glycaemic control markers such as glucose, insulin, HOMA-IR, and C-peptide levels [41]. The body of research suggests that incorporating legumes and pulses into the diet not only influences glycaemic markers but also has notable effects on insulin levels and HOMA-IR scores in individuals with type 2 diabetes. The varied outcomes across studies highlight the nuanced relationship between these foods and insulin resistance metrics.

## 4. Discussion

This study analysed 28 research articles, covering both acute postprandial trials and long-term clinical trials, including parallel and crossover designs. Distinguishing our work from others, we adopted a comprehensive approach, assimilating data from both acute and long-term trials. Further, by integrating a geographical perspective, we have offered an unprecedented view of where research efforts concentrate in relation to diabetes prevalence. Findings from the acute postprandial trials revealed that pulse and pulse-based meals consistently resulted in lower glucose responses compared to carbohydrate-matched controls. Long-term trials also indicated the benefits of incorporating pulses and legumes into the diet for better glycaemic control in T2DM patients. The research also emphasized the influence of pulse consumption on HOMA-IR scores, indicating its potential to manage insulin resistance. The collective body of evidence underscores the advantages of including legumes and pulses in the diet for T2DM patients, though the effects on insulin resistance and glycaemic markers can be multifaceted and varied across studies.

### 4.1. Pulse Composition in Diabetes Dietary Management

**Figure 3:**
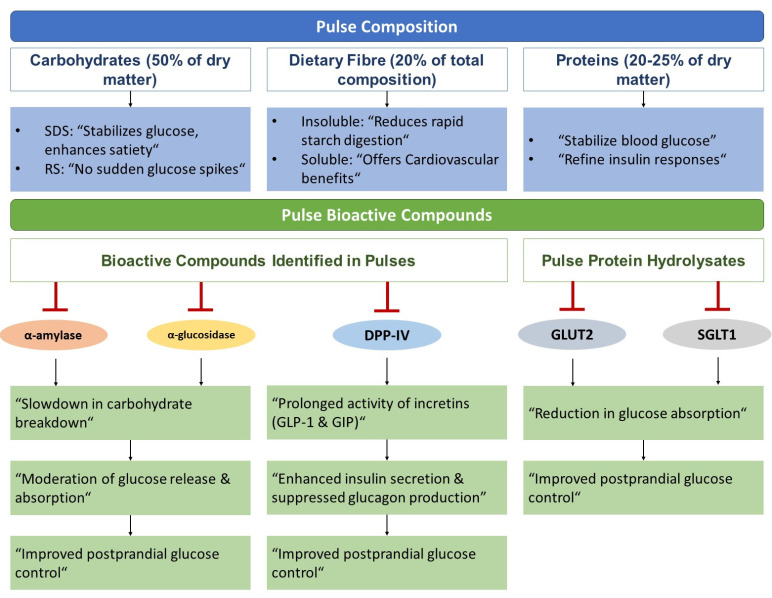
Potential mechanisms of action of pulses in the management of type 2 diabetes. SDS: slowly digestible starch; RS: resistant starch; DPP-IV: Dipeptidyl Peptidase; GLP-1: Glucagon-Like Peptide-1; GIP: Gastric Inhibitory Peptide/Glucose-dependent Insulinotropic Polypeptide; GLUT2: Glucose Transporter 2; SGLT1: Sodium Glucose Cotransporter 1.

Figure 3 illustrates the potential mechanisms by which pulses may influence the management of T2DM. Carbohydrates, an important component of pulses, make up for approximately 50% of their dry matter. Within this, starch accounts for 75–80%, accompanied by non-starch polysaccharides (i.e., dietary fibre) and smaller proportions of oligosaccharides [46,47]. Dietary starches may be classified as rapidly digestible (RDS), slowly digestible (SDS), and a fraction resistant to enzymatic digestion, known as resistant starch (RS) [48]. RDS fraction undergoes rapid digestion, resulting in swift elevations in blood glucose levels after ingestion, while the SDS is digested completely in the small intestine but at a slower rate. SDS confers particular health benefits, including stabilizing glucose metabolism resulting in improved diabetes management and enhancing satiety [49]. Resistant to enzymatic breakdown, RS does not appear to contribute to acute blood glucose spikes. Notably, the carbohydrate fraction in pulses is particularly rich in SDS and contains significant amounts of RS, a profile superior to cereals, tubers, and unripe fruits, irrespective of their processing [50,51,52]. Such a composition ensures slower starch digestion, thereby modulating postprandial glycaemic responses [51,52]. Additionally, RS may also confer an independent hypoglycaemic effect [52,53].

When compared to other fibre-rich plant-based foods, such as cereals and tubers, pulses stand out as good sources of “lente” or slow-release carbohydrates, mainly due to their higher proportions of dietary fibre [54]. Dietary fibre constitutes about 20% of the total pulse composition and can either be insoluble (75–80%) or soluble (20–25%) [47]. The presence of dietary fibre may help decrease the transit time of pulses through the small intestine [55]. The fibrous structure intrinsic to pulses impedes rapid starch digestion by limiting the access of pancreatic amylase to starch granules [56]. This restricted enzymatic access ensures a more measured and incomplete starch breakdown, which effectively lowers the glycaemic load and response associated with pulse-rich diets. Soluble fibre has also been shown to confer positive cardiovascular benefits [57], an important factor to consider in the prevention of long-term complications of diabetes. Pulses contain oligosaccharides in quantities that surpass those found in cereals [58]. These oligosaccharides may act as prebiotics, fostering a healthier gut microbiome. Additionally, they have the potential to attenuate cholesterol and triglyceride levels and amplify satiety signals, thereby indirectly aiding in weight and lipid management [59,60]. Combined with this beneficial carbohydrate composition, protein also accounts for approximately 20–25% of the dry matter of pulses [58]. Consuming adequate protein is pivotal for diabetics, as it can stabilize postprandial blood glucose levels and refine insulin responses, ensuring more effective glucose regulation [61].

### 4.2. Pulse Bioactive Compounds and Glycaemic Control

Apart from the benefits associated with their structural composition, pulses offer advantages stemming from their bioactive compounds, which are increasingly being recognized for their potential to manage blood glucose levels [62]. A few of the bioactive compounds identified in pulses are saponins, phenolics, proanthocyanins, flavonoids, anthocyanins, carotenoids, tocopherols, tannins, and bioactive peptides [63,64]. The inherent benefits of pulses in glycaemic control can be attributed to the inhibitory actions of some of these bioactive compounds on enzymes that play pivotal roles in carbohydrate digestion and glucose absorption. Specifically, components such as protein hydrolysates, certain peptide fractions, characterized bioactive peptides, phenolic compounds, phenolic acids, flavonoids, and tannins demonstrate inhibitory effects on enzymes like α-amylase, α-glucosidase, and dipeptidyl peptidase (DPP-IV) [64,65]. The inhibition of enzymes α-amylase and α-glucosidase slows down carbohydrate breakdown, leading to moderated glucose release and absorption in the small intestines [66,67]. On the other hand, DPP-IV inhibition can prolong the activity of incretins such as Glucagon-Like Peptide-1 (GLP-1) and GIP. This in turn enhances insulin secretion while simultaneously suppressing glucagon, ensuring better postprandial glucose control [64,67,68,69].

Further investigations into pulse protein hydrolysates have revealed their potential to control glucose uptake. These hydrolysates, in a dose-dependent manner, have been shown to inhibit glucose transporters Glucose Transporter 2 (GLUT2) and Sodium Glucose Cotransporter 1 (SGLT1). Such inhibitory action effectively reduces glucose absorption, as evidenced in rat model studies [64,70].

### 4.3. The Pulse Paradigm: A Dietary Revolution for Diabetic Care

Pulses present a viable solution to improved glycaemic control in diabetic diets. Their wide-based acceptance in many cultural cuisines and their versatile preparation methods lend them to being used in diverse ways in the diet. Conventionally, boiling has been the preferred method for cooking pulses. However, contemporary culinary practices have embraced baking, and the commercial sector is increasingly adopting extrusion cooking for crafting palatable pulse-based snacks [8,47]. This culinary flexibility sees pulses seamlessly fitting into a variety of dishes, from stews, soups, salads, and shakes to main dishes, or as complementary sides. Furthermore, pulse flours can be used as a nutritious and gluten-free alternative to conventional wheat flours, broadening the scope for their inclusion in myriad recipes.

Beyond their culinary appeal, the consistent consumption of pulses can enhance diabetes management. A regular pulse-rich diet can contribute to better glycaemic control, enhanced insulin sensitivity, and could potentially minimize the reliance on diabetes medications—both in terms of variety and dosage. Moreover, pulses are not merely limited to benefiting glycaemic control as their consumption has been linked to cardiovascular health improvements. Regular consumption of pulses has been correlated with improved lipid profiles and decreased triglyceride levels, subsequently reducing the risks of cerebrovascular events and myocardial infarctions [12]. This not only signifies better health outcomes for individuals but also translates to considerable savings by decreasing the global economic costs of diabetes management [71].

### 4.4. Knowledge Gaps and Suggested Research Directions

Pulses are both cultivated and consumed extensively worldwide, serving as a highly nutritious dietary resource being excellent sources of protein, carbohydrates, dietary fibre, vitamins, minerals, and phytochemicals. They are the world’s second most important legume class after soybeans and are one of the basic foods in Africa, India, and Latin America [47,72], consumed primarily in combination with cereals to help achieve daily protein requirements [9].

Over 60% of the global diabetic population is in Asia, with China and India together accounting for nearly half [73]. India and Iran, with four studies each, are at the forefront of this research despite having a diabetes prevalence of 8.3% and 9.5%, respectively. Interestingly, while China has the highest number of adults with T2DM at approximately 140.9 million and a prevalence of 13%, it has only conducted two studies, mirroring the research output from countries with significantly fewer diabetes cases such as Pakistan and the Republic of Korea. The USA, with a 13.6% prevalence rate and 32.2 million adults affected, also has only two studies to its name. Furthermore, nations like Bangladesh, Egypt, and Spain, with relatively high diabetes prevalence rates, have undertaken only a single study each. This disparity is even more pronounced when we consider countries like Kuwait and Malaysia, which have high diabetes prevalence rates of 25.5% and 20.0%, respectively [44], but lack any studies on the impact of pulse consumption in managing T2DM (Figure 2). Furthermore, regions like Latin America and the Caribbean, known for their rich pulse-centric cuisines and a rising incidence of T2DM, remain largely unexplored for the potential benefits of pulses for diabetics [4,74]. This indicates a potential knowledge gap, suggesting the need for more focused research in countries with a high burden of diabetes but limited investigations into the role of pulses in its management.

Given that developing countries represent a high global burden of diabetes, there is a pressing need for research that investigates the multifaceted benefits of pulses in their diets, such as their affordability, cultural relevance, health benefits beyond glycaemic control, and potential for sustainable agriculture. This could lend support to dietary guidelines advocating increased pulse consumption. Moreover, integrating pulses more innovatively into culinary traditions could amplify their inherent health advantages across global cuisines.

Furthermore, in many of the studies reviewed, legumes were often consumed in combination with other foods (e.g., rice or bread). The interaction between legumes and these foods can significantly influence the postprandial glycaemic response. Additionally, different preparation methods can influence the glycaemic response. For instance, soaking, boiling, or roasting can alter the digestibility and the release of sugars from legumes. Considering these factors, there is a pressing need for more detailed research. Future studies should aim to isolate the effects of legumes by examining them as a standalone dietary component, and further investigate the interactions when paired with other foods. This approach will provide a clearer picture of their true impact on glycaemic control.

### 4.5. Strengths and Limitations

This scoping review carries several notable strengths. Firstly, it was systematically conducted using a well-defined protocol based on Arksey and O’Malley’s framework, enhancing its comprehensiveness and methodological robustness. Adherence to the PRISMA-ScR guidelines underscores the thoroughness and transparency of the review process. The adoption of the Population (P), Concept (C), and Outcome (O) approach enabled a structured and targeted search strategy. Additionally, by considering articles without any publication date restriction, the review allowed for a more exhaustive assessment of the relevant literature.

What sets this study apart is our pioneering integration of a geographical analysis, offering a rare insight into the alignment between diabetes research and its global prevalence. This geomapping approach not only identifies where the majority of research efforts are localized but also highlights regions that might be underrepresented, despite a high prevalence of T2DM. Moreover, our comprehensive examination across both acute and long-term trials affords a holistic understanding, bridging gaps that might exist when these trial types are reviewed in isolation. The insights from this study also pave the way for culturally customized health promotion initiatives promoting pulse consumption in areas with high T2DM prevalence. In doing so, our review not only evaluates the role of pulses in T2DM management but also offers a blueprint for future research directions, accentuating regions and topics that need further exploration.

However, certain limitations exist. Despite the comprehensive search strategy, only articles written in English were considered, potentially overlooking valuable research in other languages. This linguistic bias may have resulted in missed evidence or an incomplete representation of global research. Furthermore, while the review provided insights from both acute postprandial trials and long-term studies, it did not differentiate the strengths of evidence based on the study design, potentially overlooking variations in methodological quality across the included studies.

## 5. Conclusions

Drawing from the data presented in the reviewed studies, it is evident that pulses play a crucial role in enhancing glycaemic control for individuals with diabetes. Acute studies demonstrate that diets rich in pulses contribute to reduced glucose, PBGR, and insulin response curves. Furthermore, meals centred around pulses consistently present with lower glycaemic indices and glycaemic loads compared to their control counterparts. Long term studies echo these benefits, revealing that sustained pulse intake significantly decreases levels of FBG, HbA1c, fasting insulin, markers of insulin resistance such as HOMA-IR, and indicators such as high C-peptide levels. Incorporating pulses into the daily diet of diabetics emerges as not only a practical and efficient method for managing the condition but also as an economically favourable approach in the broader battle against diabetes.

## Figures and Tables

**Table 1:** Inclusion Criteria.

Characteristic	Source Inclusion Criteria
Participants	Human participants diagnosed with/exhibiting symptoms of T2DM, prediabetes, or impaired glucose tolerance
Concept/Intervention	Must involve pulse crops or pulse fractions (seeds and seed fractions)
Outcomes	Modulation of glucose homeostasis in T2DM patients - Glycaemic control - Fasting glucose within normal ranges - Reduction in insulin dosage - Reduction in blood glucose fluctuations Improved quality of life Improved symptoms - Decrease in long-term adverse events/complications
Study Design	Qualitative and quantitative studies of primary or secondary research
Review Characteristics	Original research studies (not reviews) published prior to 18 July 2023 Articles written in English

**Table 2:** Characteristics of acute postprandial trials investigating the effect of pulse consumption on glycaemic indices in patients with type 2 diabetes.

Author	Study Design	Sample Size + Population	Intervention	Control	Sampling Times (min)	Outcomes	Main Findings
Jenkins et al., 1983 [16]	C	12 T2DM subjects	50 g carbohydrate portions: kidney beans, romano beans, red lentils, black-eyed peas, chickpeas	Wholemeal bread and cottage cheese meal	0, 30, 60, 90, 120, 150, 180	Glucose	For legumes, beans consistently had a lower blood glucose response when compared to other starchy foods. Kidney beans, chickpeas, and lentils exhibited the lowest blood glucose responses among the beans tested. Black-eyed peas or pinto beans did not show consistently different responses from other foods, while wholemeal and white bread, potato, cornflakes, and porridge had the highest blood glucose responses.
Tappy et al., 1986 [17]	C	4 T2DM subjects and 6 healthy subjects	Bean flake test meals	Potato flake test meal with bean fibre–protein fraction (extract)	0, 30, 60, 90, 120, 150, 180, 210, 240	Glucose, GIP	Elevation of glucose oxidation was significantly less during the 2 h after consuming beans compared to potatoes. GIP increment was significantly less at 60 min after consuming bean flakes than after potato flakes. After consuming bean flakes, glucose and insulin plasma levels increased only slightly in T2DM subjects.
Akhtar et al., 1987 [18]	C	14 T2DM subjects and 14 healthy subjects	7 test meals: bread plus egg (BE), + milk; bread plus grams (BG) + milk; Bread plus lentils (BL) + milk; bread plus moong (Bmo) + milk; bread plus mash (BMa) + milk; basmati rice plus lentils (RL) + milk; basmati rice plus moong (RMo) + milk	Bread plus egg + milk	0, 30, 60, 90, 120, 180	Glucose	Leguminous seeds from Pakistani meals resulted in a lower blood glucose response than meals with bread as the main carbohydrate source. Among the test meals, the rice and moong combination (RMo) led to the highest blood glucose response. In general, meals with beans caused lower glucose responses than bread alone.
Viswanathan et al., 1989 [19]	C	9 T2DM subjects and 9 healthy subjects	75 g carbohydrate portions: Bengal gram, black gram, green gram, red gram, masoor	75 g dextrose OGTT	0, 30, 60, 90, 120	Glucose, insulin	Legumes produced low glycaemic responses in normal controls and even lower values in diabetic patients. There was not a significant difference in glycaemic responses among the different legumes in both control and diabetic subjects. The change in insulin values (∆I) and the ratio of insulin response to glycaemic response (∆I/∆G) for legumes were lower than those produced by glucose in control subjects. In NIDDM subjects, the insulin responses to legumes were similar to those produced by glucose.
Mani et al., 1992 [20]	C	30 T2DM subjects (5 groups of 6)	Five different meals based on semolina were tested: RI: semolina alone, steamed with gelatinization; R2: semolina with black gram dhal (*Phaseolus mungo*); R3: semolina with green gram dhal (*Phaseolus aureus*); R4: semolina with Bengal gram dhal (*Cicer arietinum*); R5: semolina alone, roasted at 105 °C, which resulted in gelatinization when water was added	OGTT 50 g	0, 60, 120	Glucose	Compared to a 50 g glucose load, meals containing steam-cooked semolina and semolina–Bengal gram dhal produced a significantly lower blood glucose response at 1 h postprandially. Only meals based on semolina–black gram dhal elicited a significantly lower blood glucose response at 2 h postprandially.
Mani et al., 1994 [21]	Single-arm intervention trial (likely crossover)	20 T2DM subjects	90 g of the test meal given in the form of a ‘khichadi’ (a steam-cooked savoury preparation) made out of the cereal–pulse mix, containing 50 g carbohydrate	OGTT 50 g	0, 60, 120	Glucose	Compared to a 50 g glucose load, the test meal resulted in significantly lower blood glucose responses at both 1 h and 2 h postprandially.
Schafer et al., 2003 [22]	C	9 T2DM subjects	Meal 1: peas 100% carb (*Pisum sativum*); Meal 2: peas 2/3 carb and potatoes 1/3 carb + cooked carrots, celery, and sausages	Meal 3: potatoes (*Solanum tuberosum* var. Granola) + cooked carrots and celery, together with fried lean pork and gravy	−15, 0, 30, 90, 120, 150, 180	Glucose, insulin	Postprandial plasma glucose and insulin concentrations rose more slowly and were significantly smaller after consuming a meal based on dried peas than after one based on potatoes. Areas under the glucose curve for meals based on dried peas, a combination of peas and potatoes, and only potatoes were 164, 257, and 381 mmol·180 min/L, respectively. Meal based only on potatoes (meal 3) produced a response 3.3 times higher in glucose and 2.7 times higher in insulin at 120 min compared to the dried pea meal (meal 1).
Khan et al., 2009 [23]	C	6 T2DM subjects and 6 healthy subjects	50 g carbohydrate: mash (*Vigna mungo*), moong (*Vigna radiata*), masoor (*Lens esculenta*), chana dhal (*Cicer arietinum*) with boiled rice and chicken biryani	Bread served with egg in sunflower oil (50 g carbohydrate)	0, 30, 60, 90, 120, 150, 180	Glucose	The relative glycaemic responses for the mixed meals containing mash, moong, masoor, chana dhal, and biryani were 91%, 111%, 108%, 88%, and 101%, respectively, in normal subjects. For diabetic subjects, they were 102%, 123%, 107%, 87%, and 103%. There was no significant difference in the variation of iAUC between the test meals in both normal and diabetic subjects.
Thompson et al., 2012 [24]	C	17 T2DM subjects	Three meals included one of the commercially canned *P. vulgaris* market classes: pinto beans, black beans, or dark red kidney beans, plus ~1/2 cup of white long-grain rice	A control meal containing 180 g or approximately 7/8 cup of steamed long-grain white rice was included as the fourth meal	0, 30, 60, 90, 120, 150, 180	Glucose	Postprandial net glucose values were lower for the three bean/rice treatments in comparison to the rice control at 90, 120, and 150 min after consumption. The iAUC values for blood glucose were significantly lower for meals with pinto and black beans/rice compared to rice alone, but not for kidney beans/rice. Glucose concentrations at 90 min postprandial were significantly lower for meals with pinto beans, black beans, and red kidney beans compared to the white rice control meal. Similar significant reductions in glucose concentrations were observed at 120 and 150 min for the bean/rice meals compared to rice alone.
Alegbejo and Ameh, 2012 [25]	C	10 T2DM subjects and 6 healthy subjects	Meal: 50 g of carbohydrate made of rice, cowpea (beans), tomatoes, and palm oil with meat	50 g glucose	0, 30, 60, 90, 120, 150	Glucose	Rice/bean consumption showed a significantly lower blood glucose response from 30–150 min in diabetics. Incremental value of blood glucose following rice/bean meal in type 2 diabetics was notably lower at multiple intervals post-consumption, but not significant in healthy subjects. Diabetic subjects reached peak blood glucose response at 90 min with glucose and 60 min with rice/beans. Maximum increase in blood glucose was lower in both groups when consuming rice/beans compared to glucose. Two-hour postprandial blood glucose levels were significantly lower in both groups when consuming rice/beans compared to glucose.
Baldeón et al., 2012 [26]	Phase II Clinical Trial	30 T2DM subjects	Test meal: *Lupin mutabilis* or *Lupin mutabilis*/alkaloid extract in 2:1 ratio	NIL	0, 60, 90	Glucose, insulin	Consumption of cooked *L. mutabilis* or its purified alkaloids decreased blood glucose and insulin levels in volunteers with diabetes. The decrease in serum glucose from base line to 90 min was statistically significant within both the cooked *L. mutabilis* group and the alkaloid group. No statistically significant differences in serum glucose or insulin concentrations were observed between the two treatment groups at 0, 60, or 90 min. Subjects receiving alkaloids had a significant decrease in glucose levels at 90 min post-treatment (9.9%), while those receiving cooked *L. mutabilis* had significant glucose level reductions at both 60 and 90 min. Serum insulin concentrations did not have statistically significant changes within the groups. However, both groups saw a decrease in insulin concentrations by 90 min.
Olmedilla-Alonso et al., 2013 [27]	C	12 T2DM subjects	Beans: Almonga, Curruquilla (*P. vulgaris*)	Bread	0, 30, 60, 90, 120, 180, 240, 360	Glucose, insulin	Both bean varieties caused a similar effect on blood glucose and insulin in type 2 diabetics, but had marked differences compared to bread in terms of response magnitude and duration. There was no significant difference in glucose response between the two bean varieties. Insulin responses to both bean varieties were similar and substantially lower than the response to bread.
Olopade et al., 2020 [28]	C	14 T2DM subjects and 15 healthy subjects	Three different varieties (*V. unguiculata* [Linn Walp] varieties) of beans: *oloyin*, *drum*, *sokoto white*; 2 h boiled beans—50 g carb	50 g Glucose	0, 30, 60, 90, 120	Glucose	Among persons with T2DM: “*Oloyin*” bean meal showed the highest 2HPPG and PPG but had the lowest MIPG values compared to the other bean meals. “*Drum*” bean meal resulted in the highest MIPG and iAUC but had the lowest 2HPPG compared to the other bean meals.
Xiong et al., 2021 [29]	C	63 T2DM subjects	Bean-based diet	White rice	0, 30, 60, 120, 180	Glucose, insulin, HOMA-IR, HOMA-β, C-Peptide	Postprandial glucose levels were significantly lower in the bean-based diet group at 60, 120, and 180 min post-consumption. Insulin levels were higher at 60 min and C-peptide levels were higher at 30 and 60 min post-consumption in the bean-based diet group. The glucose AUC was significantly lower in the bean-based diet group, with no significant differences in the AUC for insulin and C-peptide (except for C-peptide AUC from 0–60 min).

C: crossover; T2DM: type 2 diabetes; GIP: gastric inhibitory peptide/glucose-dependent insulinotropic polypeptide; OGTT: oral glucose tolerance test; NIDDM: non-insulin-dependent diabetes mellitus; IAUC: incremental areas under the curves; PPG: peak plasma glucose; MIPG: maximum increase in plasma glucose; 2HPPG: 2 h postprandial glucose; IAUGC: incremental area under glucose curve; HOMA-IR: homeostatic model assessment for insulin resistance.

**Table 3:** Characteristics of long-term RCTs investigating the effect of pulse consumption on glycaemic indices in patients with type 2 diabetes.

Author	RCT Design	Sample Size + Population	Intervention	Control	Duration	Outcomes	Main Findings
Simpson et al., 1981 [30]	C	18 T2DM subjects and 9 IDDM subjects	Experimental diet (HL) high in leguminous and cereal fibre	Low-carb traditional diabetic diet (LC)	12 weeks (two 6-week periods)	Glucose, 24 h urine glucose (Glycosuria), HbA1c, plasma insulin	NIDDM Patients: The experimental high-leguminous and cereal fibre diet (HL) resulted in a significant reduction in plasma glucose during a 24 h profile compared to the control diet (LC). Basal glucose, preprandial glucose, and postprandial glucose levels were all significantly lower on the HL diet. The average glucose level over 24 h was significantly lower on the HL diet. Glycosuria was heavier on the LC diet. HbA1c was significantly lower on the HL diet. While plasma insulin levels were generally a tad lower on HL, there was no significant difference between the two diets.
Sekar et al., 2006 [31]	P	20 T2DM subjects	Modified pulse carbohydrate diet that comprised 75% pulses and 25% cereals in the form of idli or dosa	Standard diet consisting of 75% cereals and 25% pulses	12 weeks	HbA1c	HbA1c showed a significant reduction in the group consuming the modified pulse carbohydrate diet compared to the control group.
Ghattas et al., 2008 [32]	P	94 T2DM subjects	Test foods made up of traditional plants such as: Whole wheat (belila 1); Partially decorticated wheat (belila 2); Fenugreek powder seeds; Germinated fenugreek seeds; Edible lupine Roasted chickpea; Biscuit 1 (made from baked whole grain wheat and powdered fenugreek); Biscuit 2 (made from whole grain wheat and milled chickpea)	Low-caloric balanced diet	1 week	Glucose, HbA1c, insulin	There was a decrease in mean blood glucose levels at all time points in the glucose tolerance test after consuming the test foods, with significant reductions observed after consuming lupine, powdered fenugreek, partially decorticated belila (wheat), and both biscuits 1 and 2 compared to basal levels. Serum insulin levels showed a significant drop after 2 h for lupine and after consuming partially decorticated wheat (belila) after 2 h. HbA1c data indicated a numerical decrease for almost all groups, with significant reductions observed for whole wheat and biscuit 1. Lupine was an exception, showing no significant change.
Shams et al., 2008 [33]	C	30 T2DM subjects	A general diet + 50 g cooked lentil and 6 g canola oil	A general diet with some restrictions on excessive legume consumption	12 weeks (two 6-week periods)	Glucose, serum Fructosamine	Cooked lentil consumption at breakfast led to significant decreases in both total cholesterol and fasting blood glucose levels. No significant changes were observed in serum Fructosamine due to the lentil treatment.
Jenkins et al., 2012 [34]	P	121 T2DM subjects	A low-GI diet emphasizing legume consumption	A high-wheat-fibre diet emphasizing high-wheat-fibre foods	12 weeks	Glucose, HbA1c	The control group saw an increase in fasting glucose and HbA1c, while the whole grain and legume group had significant reductions in these parameters.
Kang et al., 2014 [35]	P	185 subjects with IFG or newly diagnosed T2DM	Whole grains and legumes	Refined rice	12 weeks	Glucose, insulin, HOMA-IR	A significant reduction in serum levels of glucose, triglyceride, and HOMA-IR index for all participants, irrespective of genotype. In C allele carriers, a notable decrease in insulin and an increase in HDL-cholesterol and apoA-V concentrations. In TT allele carriers, a marginal decrease in insulin and a minor rise in HDL-cholesterol and apoA-V concentrations.
Kim et al., 2014 [36]	P	99 subjects with IFG or newly diagnosed T2DM	Diet with whole grains and legumes as a carbohydrate source	A diet primarily based on refined rice	12 weeks	Glucose, HbA1c, Insulin, C-peptide, HOMA-IR	The whole grain and legume group observed significant reductions in fasting glucose, insulin, HOMA-IR index, glucose response during oral glucose tolerance tests, and HbA1c. In contrast, the control group saw increases in fasting glucose and HbA1c.
Islam et al., 2015 [37]	P	30 T2DM subjects	Bread made from composite mix flour (flour of wheat, maize, bangle gram and bean were mixed at a ratio of 35:15:25:25 to produce the composite flour mix)	Bread made from normal wheat flour	8 weeks	Glucose	FBG levels were largely non-significant in both control and intervention groups when compared to day 0, except for a significant decrease after 28 days in the intervention group using composite flour bread. The bread made from composite flour mixture significantly lowered the postprandial blood glucose level compared to normal wheat flour bread throughout the study period.
Hosseinpour-Niazi et al., 2015 [38]	C	31 T2DM subjects	Legume-based TLC diet where participants were advised to replace two servings of red meat with different types of cooked legumes like lentils, chickpeas, peas, and beans three times per week	Legume-free therapeutic lifestyle change (TLC) diet	16 weeks (two 8-week periods)	Glucose, insulin	Both diets showed decreases in FBG and fasting insulin from baseline values. Compared to the legume-free TLC diet, the legume-based TLC diet led to greater decreases in FBG and fasting insulin.
Liu et al., 2018 [39]	P	120 T2DM subjects	EABCF	Traditional LGI grains such as buckwheat, oats, barley, wheat bran, and starchy beans (excluding red adzuki beans)	4 weeks	Glucose, HbA1c, glycated albumin, insulin, HOMA-IR, HOMA-IS, HOMA-β	All four glycaemic control indicators (FBG, HbA1c, glycated albumin, fasting insulin) decreased in both groups, but no statistically significant difference was found between the groups. Subgroup analysis based on baseline fasting insulin showed that, post-intervention, there were no significant differences between the groups in HOMA-IS, HOMA-IR, and HOMA-β values.
Hassanzadeh-Rostami et al., 2019 [40]	P	75 T2DM subjects	Soybean group: A weight maintenance diet that included 2 servings of cooked soybeans 3 days a week. Legume group: A weight maintenance diet that included 2 servings of cooked non-soy legumes 3 days a week.	A weight maintenance diet including 2 servings of red meat 3 days a week	8 weeks	Glucose, insulin, HbA1c	There were significant differences at baseline in HbA1c and fasting insulin among the groups. Significant differences were observed in fasting insulin and HbA1c between groups at the end of the intervention.
Ward et al., 2020 [41]	C	22 T2DM subjects	Lupin-enriched foods consisting of multigrain bread, pasta, breakfast cereal (Weetabix™), and bread crumbs	Energy-matched wheat-based control foods	8 weeks	Glucose, insulin, C-peptide, HOMA-IR	Lupin-enriched foods had no significant impact on body weight, fasting lipids, glucose, insulin, HOMA-IR, or C-peptide levels. No significant effect on glycaemic control was noted, as measured by home blood glucose levels and both pre-meal and postprandial blood glucose readings.
Hosseinpour-Niazi et al., 2022 [42]	P	300 T2DM subjects	Hypocaloric legume-based DASH diet	Hypocaloric standard DASH diet	16 weeks	Glucose, insulin, HOMA-IR	A reduction in FBG was observed at week 16 for both dietary interventions. The legume-based DASH diet group showed greater improvement in FBG compared to the standard DASH diet group. Both dietary interventions led to reductions in insulin and HOMA-IR at week 16, with the legume-based DASH diet showing a more significant reduction in HOMA-IR.
Rebello et al., 2022 [43]	P	36 participants with insulin resistance	Low-energy-density high-pulse diet	Low-energy-density high-potato diet	8 weeks	Glucose, insulin, HOMA-IR	Both diets saw reductions in serum glucose levels, but the changes were within equivalence margins between the two diets. The bean diet showed a significant reduction in insulin response. HOMA-IR decreased in response to both diets, but the decrease was statistically significant for the bean diet.

C: crossover; P: parallel design; T2DM: type 2 diabetes; NIDDM: non-insulin-dependent diabetes mellitus; IDDM: insulin-dependent diabetes mellitus; HbA1c: haemoglobin A1c; HOMA-IR: homeostatic model assessment for insulin resistance; EABCF: extruded adzuki bean convenience food; LGI: low glycaemic index; FPG: fasting plasma glucose; HOMA-IS: homeostatic model assessment for insulin sensitivity; HOMA-β: homeostatic model assessment of beta-cell function.

## Data Availability

Not applicable.

## Supplementary Materials

The following supporting information can be downloaded at: https://www.mdpi.com/article/10.3390/nu15194222/s1, Table S1. Example strategy to identify studies on the role of pulses in the management of type 2 Diabetes Mellitus using Ovid MEDLINE.
