# Supplementary material for: Unveiling the Evidence for the Use of Pulses in Managing Type 2 Diabetes Mellitus: A Scoping Review
Source: Nutrients. 2023 Sep 30;15(19):4222. doi: 10.3390/nu15194222 (PMC10574713; doi:10.3390/nu15194222)
Supplement: Supplementary file 1 [file nutrients-15-04222-s001.zip › nutrients-2641530-supplementary.pdf]

Search Strategy
TS = consumption OR intake OR diet* OR meal OR dish* OR eat* OR ingest*
AND TS = bean OR fabaceae OR legum* OR lentil OR pulse* OR "cajanus cajan" OR "cicer arietinum" OR "cyamopsis tetragonoloba" OR dolichols OR "lathyrus sativus" OR lobia OR lupin OR phaseolus OR "pisum sativum" OR "vicia faba" OR faba OR "vigna unguiculata" OR vigna OR "bengal gram" OR "black gram" OR moong OR mung OR haricot OR feijao OR frijol OR flageolet OR dal OR dhal OR channa OR chana OR garbanzo OR kabuli OR urad OR adzuki OR afzelia OR leguminosae OR amorpha OR andira OR baptista OR callerya OR ceratonia OR clathrotropis OR colophospermum OR copaifera OR delonix OR euchresta OR guibourtia OR machaerium OR pithecellobium OR pithecolobium OR stryphnodendron OR tachigalia OR "haree matar" OR "pelee matar" OR matar OR "golden gram" or mudga OR masoor OR rajma OR "surkh lobia" OR zaragoza OR "vilayeti sem" OR habichuelas OR "coco rose" OR "rose coco" OR borlotto OR saluggia OR "tongue of fire" OR "lingua di fuoco" OR alubia OR cannellini OR "lingot mogette" OR pois OR maro OR marrowfat OR "toor dal" OR "kandhi pappu" OR "togari bele" OR "tuvaram paruppu" OR "black matpe" OR urd OR chocho OR peas
AND TS = "blood sugar levels" OR diabetes OR "glucose tolerance" OR "glucose intolerance" OR "glycemic control" OR "high blood sugar" OR hyperglycemia OR hypoglycemia OR insulin OR "insulin resistance" OR "metabolic syndrome" OR "blood glucose" OR hyperinsulinemia
NOT TS = "type 1 diabet*" OR "insulin dependent diabet*" OR "gestational diabetes" OR "pregnan*" OR "cancer" OR "bacter*" OR "soy*" OR "coffee" OR "caffeine" OR "cocoa" OR "corn" OR "tea" OR "leaf" OR "leaves" OR "pulse oximetry" OR "pulse rate" OR "pulse pressure" OR "pulse wave" OR "pulse amplitude" OR "pulsatil*" OR "myocard*" OR "muscle" OR "muscular" OR "aortic pulse" OR "atrium" OR "atria" OR "artery" OR "arterial" OR "venous" OR "ventric*" OR "peripheral puls*" OR "electr*" OR "magnet*" OR "ultrasound" OR "Mice" OR "animal" OR "mouse" OR "rat" OR "In vitro" OR "in vivo" OR "animal model" OR "rodent" OR "murine" OR "porcine" OR "sheep" OR "pigs" OR "horses" OR "cows" OR "dogs" OR "beetle" OR "poultry" OR "livestock" OR "preclinical" OR "laboratory animal" OR "animal study" OR "animal experiment" OR "animal trial" OR "animal research" OR "animal model" OR "animal testing" OR "animal physiology" OR "animal behavior" OR "veterinary" OR "zoology" OR "ethology" OR "vertebrate" OR "in vivo study" OR "in vitro study" OR "in vivo experiment" OR "in vitro experiment" "heart beat" OR "pulse generator" OR "power consumption" OR "channa punctat*" OR "zaragoza spain" OR "insulin like" OR "growth hormone"

Supplementary Table S1. Example strategy to identify studies on the role of pulses in the management of type 2 Diabetes Mellitus using Ovid MEDLINE
